# *In Vitro* Performance of Published Glypican 3-Targeting Peptides TJ12P1 and L5 Indicates Lack of Specificity and Potency

**DOI:** 10.1089/cbr.2019.2888

**Published:** 2019-10-04

**Authors:** Rose M. Berman, Olivia J. Kelada, Nicholas T. Gutsche, Raju Natarajan, Rolf E. Swenson, Ying Fu, Jessica Hong, Mitchell Ho, Peter L. Choyke, Freddy E. Escorcia

**Affiliations:** ^1^ Molecular Imaging Program, Radiation Oncology Branch, National Cancer Institute, National Institutes of Health, Bethesda, Maryland.; ^2^ In Vivo Imaging, Discovery and Analytics, PerkinElmer, Inc., Hopkinton, Massachusetts.; ^3^ Imaging Probe Development Center, National Heart, Lung and Blood Institute, National Institutes of Health, Bethesda, Maryland.; ^4^ Laboratory of Molecular Biology, National Cancer Institute, National Institutes of Health, Bethesda, Maryland.

**Keywords:** GPC3, hepatocellular carcinoma, peptide

## Abstract

***Background:*** Glypican 3 (GPC3), a plasma membrane heparan sulfate proteoglycan, is overexpressed on human hepatocellular carcinoma and may represent a promising biomarker. Several studies have reported peptides that selectively bind to GPC3 and could serve as scaffolds for imaging or therapeutic agents.

***Materials and Methods:*** We synthesized variants of two previously published peptides, DHLASLWWGTEL (TJ12P1) and RLNVGGTYFLTTRQ (L5), and evaluated their *in vitro* binding performance in paired isogenic cell lines, A431(GPC3^−^) and A431-GPC3^+^ (G1), as well as the liver cancer cell line HepG2. Using flow cytometry and biolayer interferometry (BLI), we compared the binding of the TJ12P1 and L5 peptide variants to the binding of corresponding scrambled peptides having the same amino acid composition, but in random sequence.

***Results:*** While both peptides bound to G1 and HepG2, they also bound to A431. The corresponding scrambled peptides demonstrated greater apparent binding to both G1 and A431 than their specific counterparts. BLI confirmed lack of binding at 0.5–1 μM for both peptides.

***Conclusions:*** We conclude that neither TJ12P1 nor L5 variant demonstrates selectivity for GPC3 at concentrations near the reported *K*_D_, and that the peptides lack potency or are nonspecific, making them inadequate for use as imaging agents.

## Introduction

Hepatocellular carcinoma (HCC) represents about 90% of all cases of primary liver cancer, ranking as the sixth most common neoplasm and the third leading cause of cancer death worldwide.^1,2^ In contrast to trends of other malignancies in the United States, HCC incidence and mortality are increasing.^3^ Diagnosis and surveillance of HCC typically involve ultrasound, computed tomographic, and/or magnetic resonance imaging. Treatment can range from transplant, surgical resection, or local ablative therapies such as radiofrequency ablation, transarterial embolization, or stereotactic radiotherapy.^4^ Systemic agents such as tyrosine kinase inhibitors (e.g., sorafenib and lenvatinib) can be deployed in the more advanced setting.^5,6^ Despite several options for interventions, no truly tumor-selective imaging or therapeutic agents exist for clinical use.

Glypican 3 (GPC3) is a plasma membrane heparan sulfate proteoglycan that is both overexpressed in and characteristic of HCC, suggesting it may be a promising tumor-selective biomarker.^7^ Several full-length antibodies, antibody fragments, and peptides for imaging and/or therapeutic use have been developed for GPC3 with varying degrees of success (Table 1).^8–17^ While full-length antibodies and antibody derivatives have demonstrated apparent *in vivo* localization, we were interested in evaluating small molecules to use as imaging scaffolds due to the potential for same-day imaging and, perhaps, improved tumor penetration.^13^ Of the published GPC3-selective peptides, they selected DHLASLWWGTEL (TJ12P1) and RLNVGGTYFLTTRQ (L5) due to their comparatively low reported dissociation constants (K_D_). Zhu et al. reported the *K*_D_ of Cy5.5-TJ12P1 to be 390.03 ± 27.47 nM.^9^ Although the original article describing L5 did not report a *K*_D_, Wang et al. found the *K*_D_ of the unmodified peptide to be 44.7 nM through surface plasmon resonance.^10,11^

**Table 1.** A List of Published Peptides and Other Anti-Glypican 3-Targeting Moieties

*GPC3-targeting molecule*	*Humanized*	*K_D_ (nM)*	*References*
DHLASLWWGTEL (TJ12P1)	N/A	390 (with Cy5.5)	^9^
RLNVGGTYFLTTRQ (L5)	N/A	44.7	^7,10^
DYEMHLWWGTEL (IPA)	N/A	225.1	^14^
THVSPNQGGLPS (GBP)	N/A	753	^15^
hGC33	Yes	0.67	^16^
YP7 (hYP7)	Yes	0.3^a^	^12^
HN3	Yes	0.6	^8^
HS20	Yes	0.3	^17^
αGPC3-F(ab′)	No	0.03	^13^

^a^ *K*_D_ value is for the nonhumanized version of the antibody, as no *K*_D_ was reported for the humanized variant (hYP7).

GPC3, Glypican 3.

N/A, not applicable.

In this study, we synthesize unlabeled and sulfo-Cy5-conjugated variants of TJ12P1 and L5 to assess their ability to bind specifically to GPC3 in cell-based and cell-free assays.

Typically, peptides used successfully as molecular imaging agents require *K*_D_ or IC_50_ values below 20 nM.^18–22^ Given that the reported binding affinities of both peptides were much higher than this threshold, our goal was to first confirm their GPC3 binding *in vitro* at concentrations near their reported *K*_D_ values and then alter the peptide sequences to develop higher-affinity, peptide-based imaging probes for HCC and other GPC3-expressing cancers.

## Materials and Methods

### Cell culture

A431, a human epidermoid carcinoma GPC3^−^ cell line, and A431-GPC3^+^ (G1), a transfected A431 cell line engineered to stably express human GPC3, were both obtained from Dr. M.H. (National Cancer Institute, Bethesda, MD).^12^ HepG2 (GPC3^+^ liver cancer cell line) was obtained from ATCC (Manassas, VA). All cell lines were cultured in DMEM (Life Technologies, Carlsbad, CA) supplemented with 10% FetaPlex (Gemini Bio-Products, West Sacramento, CA) according to supplier's protocols. All cell lines tested negative for mycoplasma in monthly tests and were used for experiments within 10 passages (HepG2) or 15 passages (A431 and G1).

### Peptide synthesis

The Imaging Probe Development Center (National Heart, Lung, and Blood Institute, Frederick, MD) synthesized fluorescent and non-fluorescent variants of both peptide sequences, DHLASLWWGTEL (TJ12P1) and KKK-RLNVGGTYFLTTRQ (KKK-L5), as well as two scrambled variants, WLSHLGDLTWEA (TJ12P1 scramble) and KKK-YTRFLGTVGNRLTQ (KKK-L5 scramble), as a control and nonspecific binding blocking agents. TJ12P1 was first synthesized with Cy5.5 as the fluorophore, as per Zhu et al., but due to poor solubility in aqueous solution, a sulfo-Cy5-labeled variant was synthesized and used for experiments.^9^ The four peptides—TJ12P1, KKK-L5, and their two scrambled variants—were synthesized with and without sulfo-Cy5 (Supplementary Table S1 and Figures S1,S2,S3,S4).

### Flow cytometry

Approximately 1 × 10^6^ cells per sample tube were harvested, washed once with ice-cold staining buffer (1% bovine serum albumin in 1 × phosphate buffered saline [PBS]), and resuspended in 60 μL staining buffer. Cells were then distributed into control and experimental vials. Unstained and single-color control samples were made. Experimental samples were stained with PE-conjugated anti-GPC3 antibody (R&D Systems, Minneapolis, MN), PE-conjugated IgG2 isotype control (Biolegend, San Diego, CA), conjugated specific or nonspecific peptide and incubated for 1–2 h on ice in the dark. Data were collected using a BD FACSCalibur cytometer running BD CellQuest Pro software (v6.0), and results were analyzed with FlowJo (v10.4.2). All experiments represent biological replicates (*n* = 3).

### Biolayer interferometry

Biotinylated human GPC3 protein (Acro Biosystems, Newark, DE) was diluted to 5 μg/mL in assay buffer (1 × PBS with 0.02% Tween-20) in a 96-well plate and loaded onto streptavidin biosensors (FortéBio, Menlo Park, CA). Each unconjugated peptide was diluted in assay buffer at four concentrations (1000, 500, 250, and 125 nM for TJ12P1; and 500, 250, 125, and 62.5 nM for KKK-L5) and loaded into the 96-well plate in a final volume of 200 μL. Specific and nonspecific binding wells were made for each concentration. The plate was run on an Octet Red96 system (FortéBio) and analyzed with FortéBio Octet Data Analysis software (v.11).

## Results

**FIG. 1.**
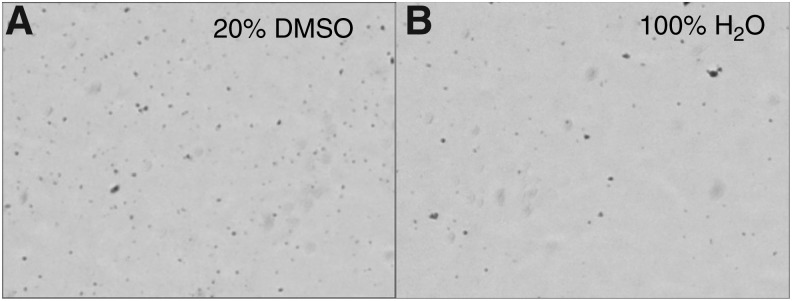
Cy5.5-conjugated TJ12P1 is peptide insoluble in aqueous buffer. Bright field images of Cy5.5-TJ12P1 peptides in solution. In **(A**, **B),** peptide Cy5.5-TJ12P1 is undissolved in 20% DMSO/80% ddH_2_O and 100% ddH_2_O at 0.3 mg/mL, respectively. DMSO, dimethyl sulfoxide.

Solubility assays were performed to determine the most compatible solvent for both peptides before *in vitro* evaluation of GPC3 binding, favoring aqueous solvents over cytotoxic organic solvents such as dimethylformamide (DMF) and dimethyl sulfoxide (DMSO). While Zhu et al. indicated that Cy5.5-TJ12P1 was soluble in DMF at the synthesis stage, authors did not report the formulation for *in vivo* studies.^9^ We found this peptide (at 0.3 mg/mL) to be insoluble in ddH_2_O (Fig. 1A) and ddH_2_O + 20% DMSO (Fig. 1B), as evidenced by the suspension of blue powder debris in solvent. Sonication and vortexing of samples failed to solubilize the Cy5.5-TJ12P1 powder, and the percent of DMSO was not increased due to the potential for biological toxicity.^24^ The sulfo-modified variant, sulfo-Cy5-TJ12P1, appeared to be soluble in 0.15 M NH_4_OAc. Han *et al*. reported that a variant of the L5 peptide with three lysine residues (KKK-L5) had higher solubility in aqueous solution compared to the original L5 peptide, so this KKK-L5 variant was synthesized^23^ and sulfo-Cy5-KKK-L5 were both soluble in ddH_2_O at 0.5 mg/mL.

**FIG. 2.**
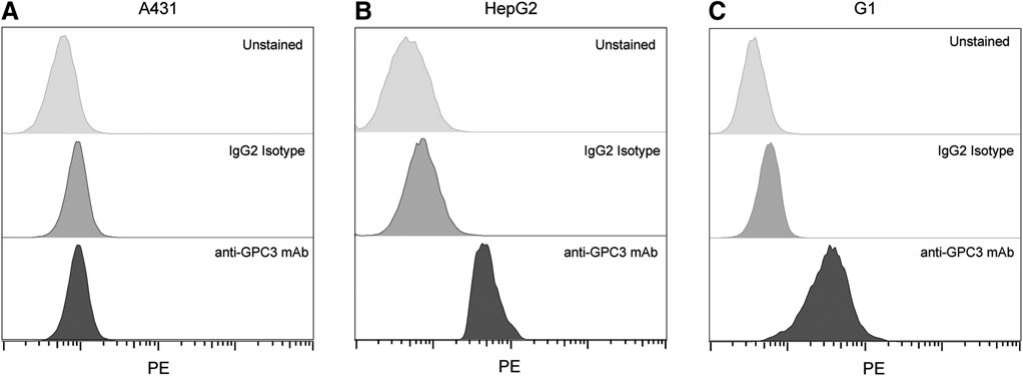
Commercial anti-GPC3 antibody confirms expression of GPC3. Flow cytometry histograms confirming that **(A)** A431 cell lines do not express GPC3 and that **(B)** HepG2 and **(C)** G1 do.

To confirm the expression of GPC3 in GPC3^+^ cell lines (G1 and HepG2), we tested cell binding with flow cytometry. The control A431 cells incubated with buffer, PE anti-GPC3 antibody, or PE IgG2 isotype control antibody demonstrated comparable staining, confirming that A431 does not express GPC3 (Fig. 2A). In contrast, HepG2 (Fig. 2B) and G1 (Fig. 2C) showed a rightward shift of the histogram in the PE channel compared to controls, confirming GPC3 expression in these two cell lines. Because G1 is derived from A431, these two lines are otherwise isogenic and serve as ideal positive and negative controls for GPC3 expression, respectively.

**FIG. 3.**
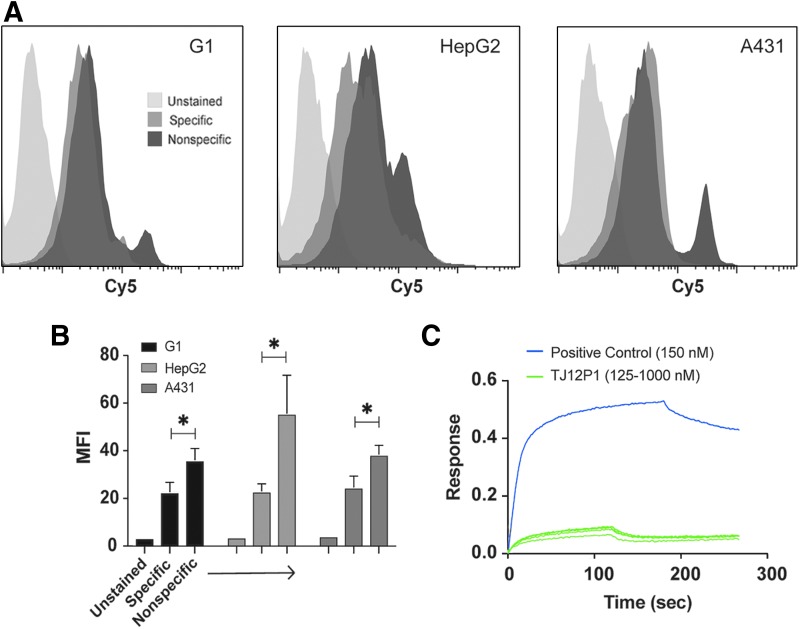
TJ12P1 binds nonspecifically. **(A)** Flow cytometry histograms of GPC3^+^ (G1 and HepG2) and GPC3^−^ (A431) cells incubated with 325 nM of sulfo-Cy5-TJ12P1 for 1 h demonstrating nonspecific association of TJ12P1 to all cell lines. **(B)** *Bar* graphs of MFI values for all cell lines ether unstained or incubated with 325 nM of the specific (sulfo-Cy5-TJ12P1) or nonspecific (TJ12P1 scramble) for 1 h indicating that nonspecific peptide had more binding to all cell lines tested (*p* < 0.05). **(C)** Biolayer interferometry response curve shows the association and dissociation of TJ12P1 peptide to recombinant GPC3 at various concentrations (125–1000 nM) compared to a known GPC3-specific molecule (*K*_D_ of 4–6 nM) at 150 nM. TJ12P1 failed to demonstrate concentration-dependent binding behavior consistent with normal, specific equilibrium binding, and no *K*_D_ could be calculated. MFI, mean fluorescence intensity. * indicates *p* < 0.05.

We then performed analogous experiments with sulfo-Cy5-TJ12P1 and its scrambled variant (sequence reported above). Results showed no significant difference in binding between G1, HepG2, and A431 cells after incubation with sulfo-Cy5-TJ12P1 at 325 nM (reported *K*_D_ = 390 nM) (Fig. 3A; Table 1).^9^ Surprisingly, the sulfo-Cy5-TJ12P1 scrambled peptide at the same concentration demonstrated significantly increased binding in all GPC3^+^ cell lines compared to the sulfo-Cy5-TJ12P1 peptide (*p* = 0.02) (Fig. 3B). To rule out the possibility that fluorophore modification of TJ12P1 interfered with its ability to specifically bind GPC3, they performed a cell-free, label-free biolayer interferometry (BLI) assay. BLI-derived association and dissociation curves of unlabeled TJ12P1 at various concentrations to immobilized recombinant biotinylated GPC3 failed to demonstrate concentration-dependent binding behavior consistent with normal, specific equilibrium binding, and no *K*_D_ could be calculated. In contrast, a known GPC3-specific antibody (*K*_D_ of 4–6 nM) at 150 nM showed *k*_on_ and *k*_off_ curves consistent with specific binding, and the software was able to confirm the *K*_D_ (Fig. 3C). Although binding curves are somewhat dependent on molecular weight, and TJ12P1 and the positive control molecule differ significantly in this parameter, the association/dissociation curves of TJ12P1 on their own indicate the absence of concentration-dependent binding.

**FIG. 4.**
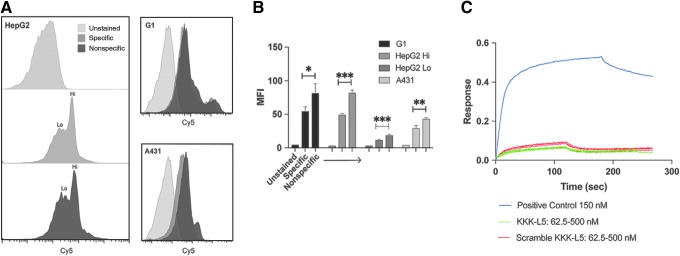
L5 variant binds nonspecifically. **(A)** Flow cytometry histograms show the Cy5 emission shift in GPC3^+^ HepG2 (Lo and Hi were used to differentiate the two HepG2 cell populations with different extent of peptide association) and G1 cells compared to GPC3^−^ A431 cells. **(B)** *Bar* graph shows MFI values for all cell lines ether unstained or incubated with 300 nM of the specific (sulfo-Cy5-KKK-L5) or nonspecific (sulfo-Cy5-KKK-L5 scramble) for 1 h. MFI values suggest that the nonspecific peptide binds better to all cell lines than the specific peptide. *p < 0.05, **p < 0.005, ***p < 0.0001. **(C)** Biolayer interferometry response curve shows the association and dissociation of KKK-L5 and KKK-L5 scramble to recombinant human GPC3 at various concentrations (62.5–500 nM) compared to a known GPC3-specific molecule (*K*_D_ of 4–6 nM) at 150 nM. KKK-L5 failed to demonstrate concentration-dependent binding behavior consistent with normal, specific equilibrium binding, and no *K*_D_ could be calculated.

Flow cytometry assays of sulfo-Cy5-KKK-L5 indicate that the peptide bound nonspecifically to GPC3, as the observed histogram shift is identical in the A431 (GPC3^−^) and the HepG2 and G1 (GPC3^+^) cells (Fig. 4A). In addition, the nonspecific peptide (sulfo-Cy5-KKK-L5 scramble) exhibited more binding (*p* < 0.05) than the specific (sulfo-Cy5-KKK-L5) peptide in all three cell lines. The high- and low-staining HepG2 populations both showed statistically significant (*p* < 0.001 and *p* < 0.005) difference in staining with the nonspecific peptide compared to sulfo-Cy5-KKK-L5 (Fig. 4B). A BLI analysis of the unlabeled KKK-L5 peptide at various concentrations also confirmed absence of specific binding to GPC3 at these concentrations. The nonspecific peptide at the same concentrations performed comparably (Fig. 4C). These results were in contrast to the performance of the known GPC3-specific antibody.

## Discussion

GPC3 is a promising HCC-selective biomarker, and a number of groups have exploited this feature to generate vaccines, HCC-selective antibodies, and peptides for imaging and therapy (Table 1).^25^ Several peptides with putative specificity to GPC3 have been reported in the literature. While TJ12P1 and L5 have emerged as the most promising peptides based on published binding affinities, in this study, we demonstrate that neither fluorescently labeled nor unlabeled versions tested can bind to GPC3 at concentrations in the range of their published *K*_D_ (0.3–1 μM).^9–11^

Previous studies investigating TJ12P1 and L5 have some limitations, notably absent controls for nonspecific binding on cells,^14^ the comparison of nonisogenic cell lines,^26^ and the incubation of cells with peptide concentrations well above the reported binding affinities (10–20 μM).^10,26^ Without using cell lines that only differ in expression of the target of interest to control for off-target associations, or scrambled peptide controls to account for nonspecific peptide-cell interactions, it is difficult to conclude any associations are specific to a target of interest. In the absence of our evaluation of both peptides and their scrambled versions in the A431 cell line (GPC3^−^), to which all peptide variants bound, we may have reasonably concluded that the scrambled peptides were improvements on the originals, as suggested by significantly improved staining of G1(A431-GPC3^+^) and HepG2 cells on flow cytometry. These findings underscore the challenges of peptide engineering and the need for employing multiple assays to corroborate specific binding, as well as appropriate controls to avoid confirmation bias.

The relative hydrophobicity of both peptides may have contributed to their nonspecific binding and makes them suboptimal translational candidates in their current forms even if they had exhibited potent, specific binding. It is also important to note that the unexpected nonspecific (or nonpotent) binding of TJ12P1 may be explained by the similarity of its sequence to that of peptides found to nonspecifically bind polystyrene walls—a common labware plastic. TJ12P1 was identified by phage panning, a process that can generate specific peptides as well as “target-unrelated” peptides (TUPs) that bind to components of the screening system such as polystyrene, streptavidin, or bovine serum albumin. Like TJ12P1, many of these TUPs contain two consecutive tryptophan residues.^27^ Bakhshinejad et al. suggest depleting the phage library through subtractive biopanning (e.g., by affinity chromatography or screening against other cell types) to reduce selection of TUPs.^27^ Isogenic cell-based assays could also prove valuable.

In contrast to TJ12P1, L5 was identified by a pull-down immunoprecipitation assay and matrix-assisted laser desorption/ionization-time-of-flight mass spectrometry analysis.^10^ Four out of the 14 residues are hydrophobic (28.5%), the presence of which may have contributed to the nonspecific binding of L5 during the selection process.

This study has a few limitations. First, as we were focused on identifying peptides useful at concentrations appropriate for imaging (*K*_D_ <20 nM), we did not test either peptide at concentrations in the high micromolar range, unlike several authors of prior publications. Although one can conclude that TJ12P1 and KKK-L5 do not bind specifically at concentrations in the range of their reported *K*_D_, we cannot rule out the possibility that the peptides may have some specificity, but are simply not potent. In addition, their study did not evaluate the original L5 peptide due to the reportedly poor solubility, but rather synthesized and tested the KKK-L5 to be able to use a biocompatible buffer for cell-based studies.

Nevertheless, given the uncompelling performance with regard to the specificity and potency of TJ12P1 and L5 variants in the assays, as well as their hydrophobicity, authors do not recommend further developing the current form of either peptide as a molecular imaging agent.

## Conclusions

The overexpression of GPC3 in HCC represents an opportunity to develop new imaging and therapeutic drugs for this disease, and a number of groups are working toward this goal. In this study, we assessed the GPC3 binding performance of variants of two published peptides, TJ12P1 and L5, with purported specificity to GPC3. At concentrations in the range of the reported *K*_D_, the peptides demonstrate equivalent binding to GPC3^+^ cells and GPC3^−^ cells, and lower binding to all cell lines when compared with nonspecific peptide controls. Furthermore, in cell-free, label-free assays, neither peptide exhibited characteristics suggestive of specificity to GPC3 at submicromolar concentrations. Coupled with poor solubility in aqueous solutions and lack of demonstrable specificity and potency of TJ12P1 and L5 for GPC3, we conclude that neither is appropriate for development as molecular imaging agents.

## Supplementary Material

Supplemental data

Supplemental data

Supplemental data

Supplemental data

Supplemental data
