# Supplementary material for: In Vitro Performance of Published Glypican 3-Targeting Peptides TJ12P1 and L5 Indicates Lack of Specificity and Potency
Source: Cancer Biother Radiopharm. 2019 Oct 4;34(8):498–503. doi: 10.1089/cbr.2019.2888 (PMC6802730; doi:10.1089/cbr.2019.2888)
Supplement: Supplemental data [file Supp_FigureS4-TableS1.pdf]

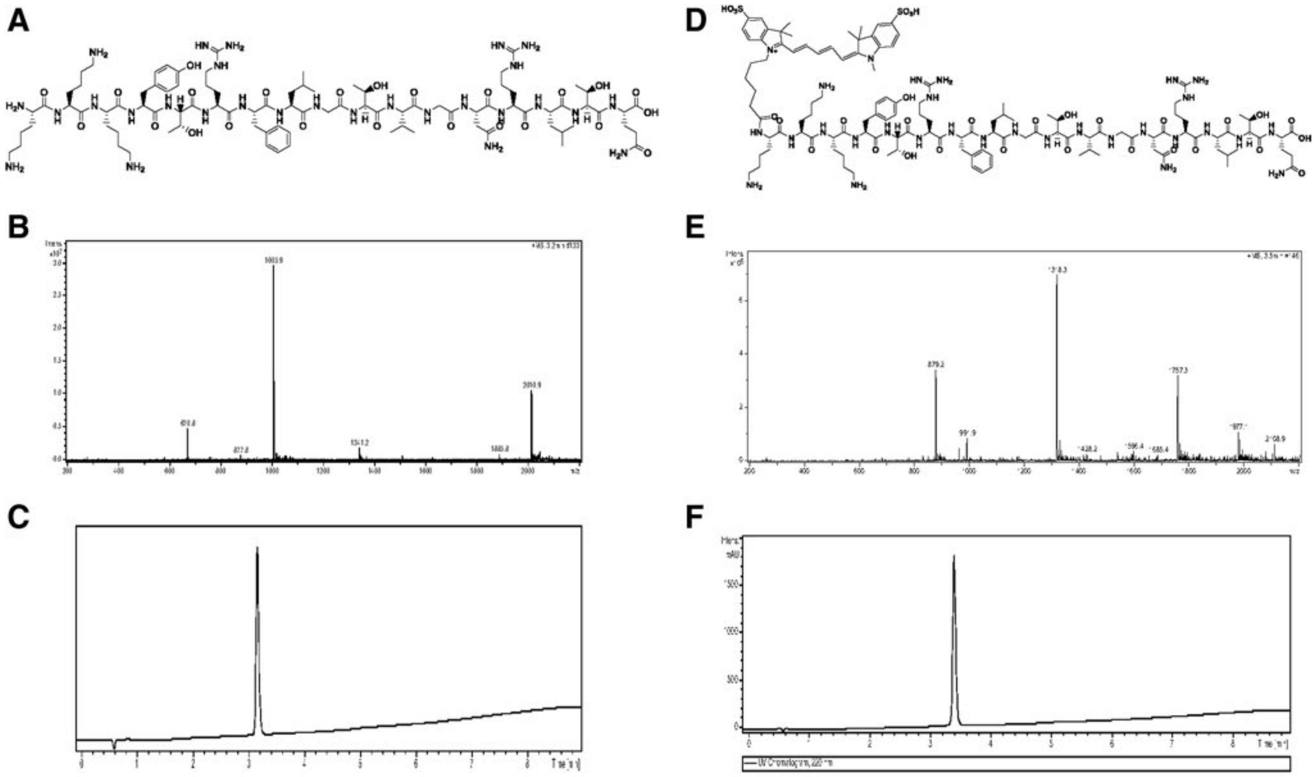

SUPPLEMENTARY FIG. S4. Characterization of KKKYTRFLGTVGNRLTQ (scrambled KKK-L5) and sulfo-Cy5-KKKYTRFLGTVGNRLTQ. **(A)** Chemical structure of Sulfo-Cy5-KKKYTRFLGTVGNRLTQ (sequence: KKKYTR FLGTVGNRLTQ). Chemical formula: $C_{122}H_{189}N_{30}O_{31}S_2$, Molecular weight: 2636.15 g/mol. **(B)** Mass Spec of Sulfo-Cy5-KKKYTRFLGTVGNRLTQ, multiple ions were detected; of these, the dominant peak was the ion [Sulfo-Cy5-KKKY TRFLGTVGNRLTQ]2+ (*m/z* 1318.3). **(C)** Analytical HPLC chromatogram of Sulfo-Cy5-KKKYTRFLGTVGNRLTQ. Retention time (t_R) = 3.50 min using a binary H_2O/ACN mobile phase containing 0.05% TFA. **(D)** Chemical structure of scrambled KKK-L5 (sequence: KKKYTRFLGTVGNRLTQ). Chemical formula: $C_{90}H_{152}N_{28}O_{24}$, Molecular weight: 2010.38 g/mol. **(E)** Mass Spec of KKKYTRFLGTVGNRLTQ, multiple ions were detected, two ions were detected as being most dominant, those of [KKKYTRFLGTVGNRLTQ]2+ (*m/z* 1005.9) and $[KKKYTRFLGTVGNRLTQ]^+$ (*m/z* 2010.9). **(F)** Analytical HPLC chromatogram of KKKYTRFLGTVGNRLTQ. Retention time (t_R) = 3.20 min using a binary H_2O/ACN mobile phase containing 0.05% TFA.

Supplementary Table S1. List of Peptide Sequences and the Associated Cleavage Procedures and Yields

Sequence	*Cleavage procedure*	*Yield*
Sulfo-Cy5-DHLASLWWGTEL	A and C	10%; blue solid
DHLASLWWGTEL	B	24.4%; colorless solid
Sulfo-Cy5-WLSHLGDLTWEA (scrambled)	A and C	11%; blue solid
WLSHLGDLTWEA (scrambled)	B	23.2%; colorless solid
Sulfo-Cy5-KKKRLNVGGTYFLTTRQ	A and C	13%; blue solid
KKKRLNVGGTYFLTTRQ	B	30%
Sulfo-Cy5-KKKYTRFLGTVGNRLTQ (scrambled)	A and C	11.5%; blue solid
KKKYTRFLGTVGNRLTQ (scrambled)	B	28%; colorless solid
