# Supplementary material for: In Vitro Performance of Published Glypican 3-Targeting Peptides TJ12P1 and L5 Indicates Lack of Specificity and Potency
Source: Cancer Biother Radiopharm. 2019 Oct 4;34(8):498–503. doi: 10.1089/cbr.2019.2888 (PMC6802730; doi:10.1089/cbr.2019.2888)
Supplement: Supplemental data [file Supp_FigureS1.pdf]

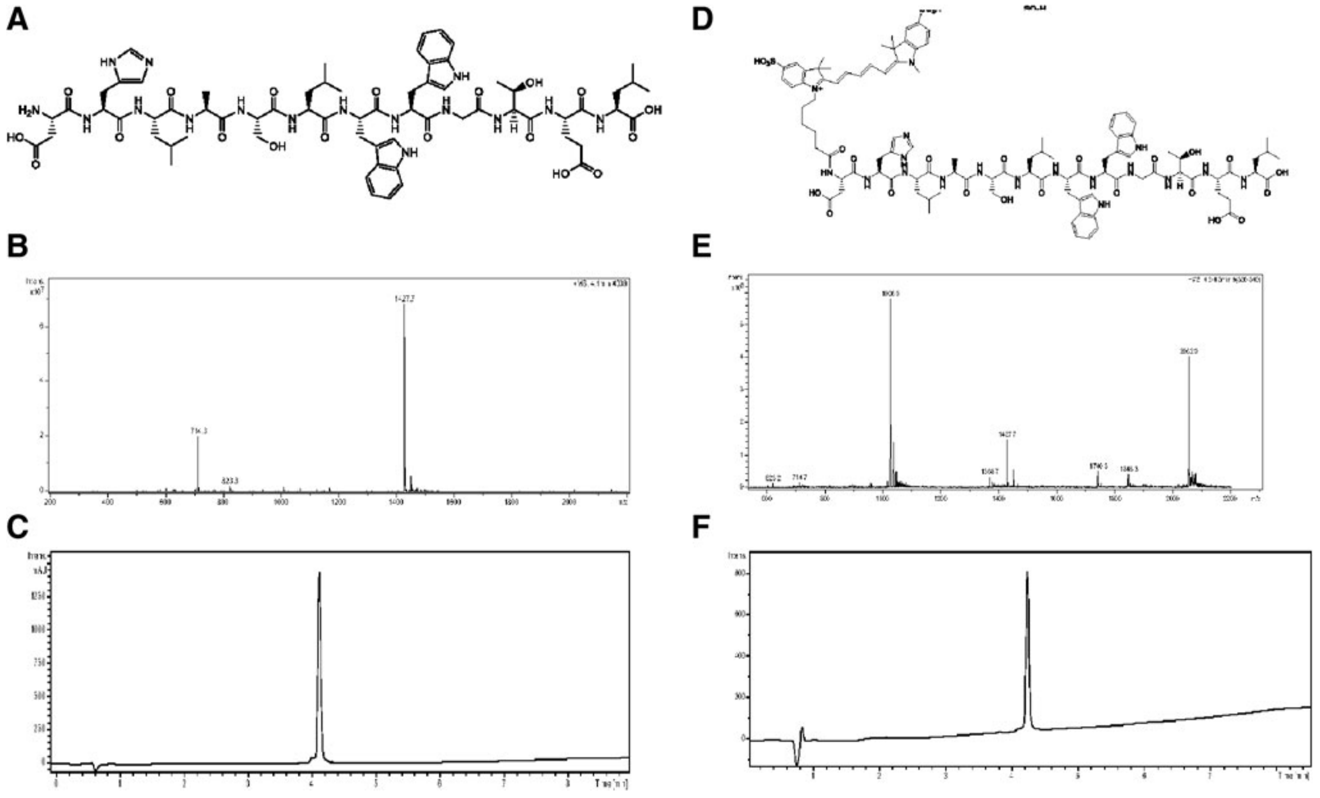

SUPPLEMENTARY FIG. S1. Characterization of TJ12P1 and sulfo-Cy5-TJ12P1. **(A)** Chemical structure of TJ12P1 (sequence: DHLASLWWGTEL). Chemical formula: $C_{67}H_{95}N_{17}O_{1}$, Molecular weight: 1426.60 g/mol. **(B)** Mass Spec of TJ12P1, two ions were detected, those of [TJ12P1]2+ (*m/z* 714.3) and $[TJ12P1]^{+}$ (*m/z* 1427.7). **(C)** Analytical HPLC chromatogram of TJ12P. Retention time $(t_R) = 4.18$ min using a binary H_2O/ACN mobile phase containing 0.05% TFA. **(D)** Chemical structure of sulfo-Cy5-TJ12P1. Chemical formula: $C_{99}H_{132}N_{19}O_{25}S_2^{+}$, Molecular weight: 2052.37 g/mol. **(E)** Mass Spec of sulfo-Cy5-TJ12P1, multiple ions were detected with the two dominant peaks of the spectrum being [sulfo-Cy5-TJ12P1]2+ (*m/z* 1026.9) and $[\text{sulfo-Cy5-TJ12P1}]^{+}$ (*m/z* 2052.9). **(F)** Analytical HPLC chromatogram of sulfo-Cy5-TJ12P1. Retention time $(t_R) = 4.22$ min using a binary H_2O/ACN mobile phase containing 0.05% TFA. HPLC, high-performance liquid chromatography; TFA, trifluoroacetic acid.
