# Supplementary figures and images for: In Vitro Performance of Published Glypican 3-Targeting Peptides TJ12P1 and L5 Indicates Lack of Specificity and Potency
Source: Cancer Biother Radiopharm. 2019 Oct 4;34(8):498–503. doi: 10.1089/cbr.2019.2888 (PMC6802730; doi:10.1089/cbr.2019.2888)

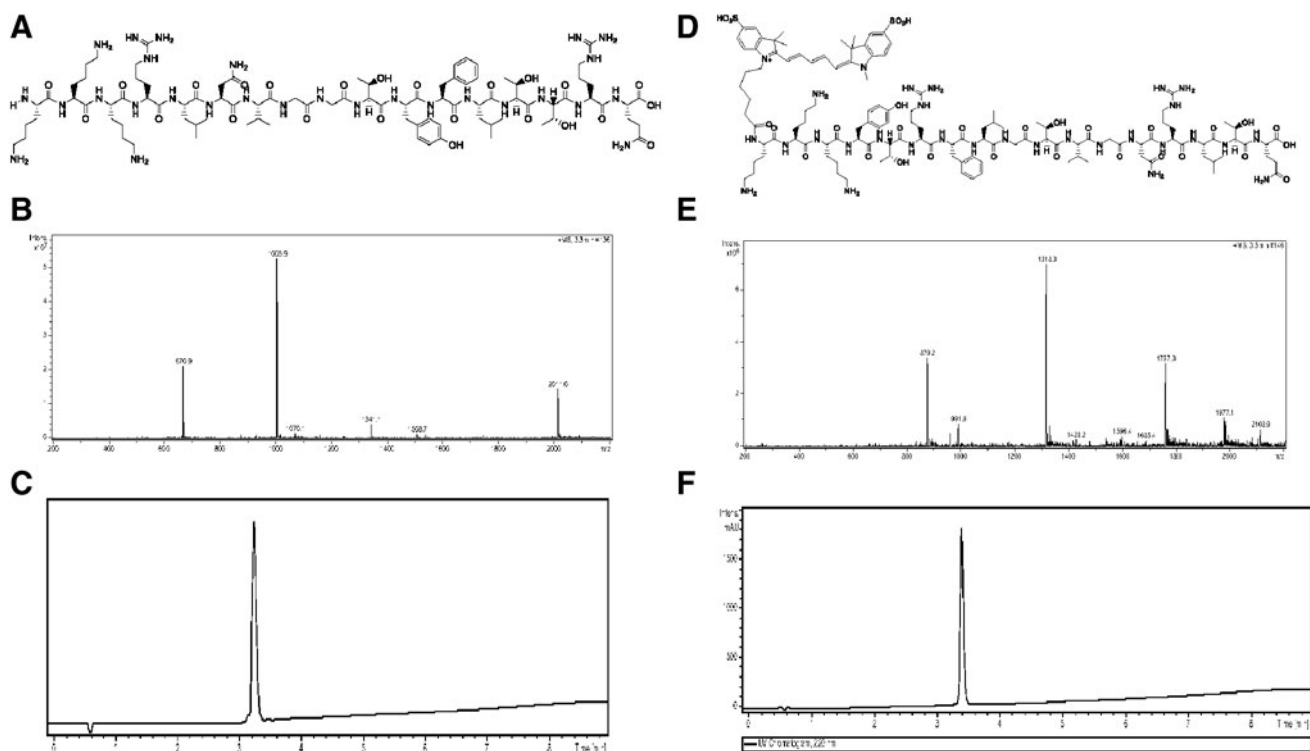

Supplement: Supplemental data [file Supp_FigureS2.pdf]
