# Supplementary material for: In Vitro Performance of Published Glypican 3-Targeting Peptides TJ12P1 and L5 Indicates Lack of Specificity and Potency
Source: Cancer Biother Radiopharm. 2019 Oct 4;34(8):498–503. doi: 10.1089/cbr.2019.2888 (PMC6802730; doi:10.1089/cbr.2019.2888)
Supplement: Supplemental data [file Supp_FigureS3.pdf]

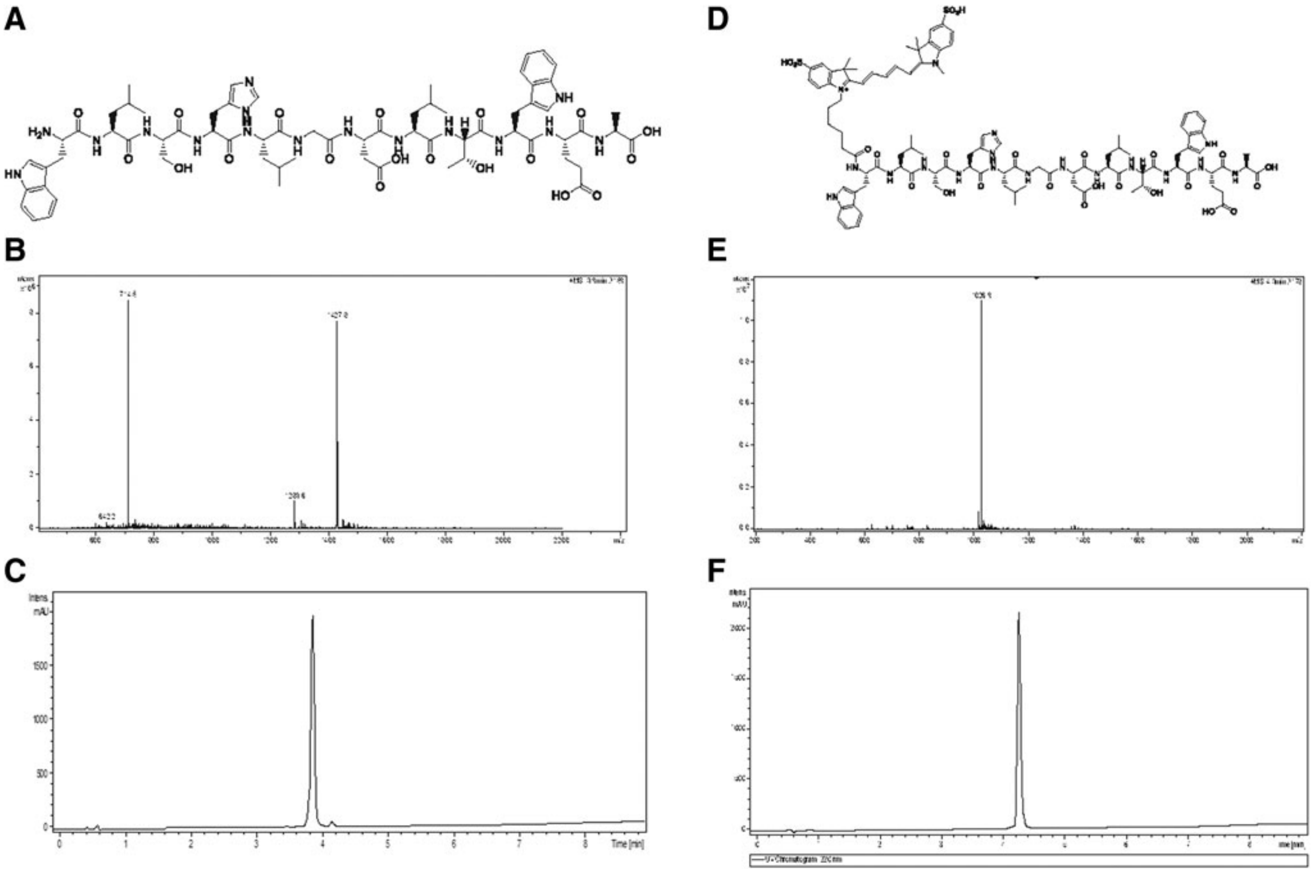

SUPPLEMENTARY FIG. S3. Characterization of WLSHLGDLTWEA (scrambled TJ12P1) and sulfo-Cy5-WLSHL GDLTWEA. (**A**) Chemical structure of sulfo-Cy5-TJ12P1 scramble (sequence: WLSHLGDLTWEA). Chemical formula: $C_{99}H_{131}N_{18}O_{26}S_2$, Molecular weight: 2053.36 g/mol. (**B**) Mass Spec of sulfo-Cy5-WLSHLGDLTWEA, a single species was detected, namely, the ion [sulfo-Cy5-WLSHLGDLTWEA]2+ (*m/z* 1026.9). (**C**) Analytical HPLC chromatogram of sulfo-Cy5-WLSHLGDLTWEA. Retention time (t_R) = 4.30 min using a binary H_2O/ACN mobile phase containing 0.05% TFA. (**D**) Chemical structure of Cy5-TJ12P1 scramble (sequence: WLSHLGDLTWEA). Chemical formula: $C_{67}H_{94}N_{16}O_{19}$, Molecular weight: 1427.58 g/mol. (**E**) Mass Spec of WLSHLGDLTWEA, two ions were detected, those of [WLSHLGDLTWEA]2+ (*m/z* 714.5) and $[WLSHLGDLTWEA]^+$ (*m/z* 1427.8). (**F**) Analytical HPLC chromatogram of WLSHLGDLTWEA. Retention time (t_R) = 3.90 min using a binary H_2O/ACN mobile phase containing 0.05% TFA.
