# Supplementary material for: In Vitro Performance of Published Glypican 3-Targeting Peptides TJ12P1 and L5 Indicates Lack of Specificity and Potency
Source: Cancer Biother Radiopharm. 2019 Oct 4;34(8):498–503. doi: 10.1089/cbr.2019.2888 (PMC6802730; doi:10.1089/cbr.2019.2888)
Supplement: Supplemental data [file Supp_Data.pdf]

Supplementary Data

Supplementary Materials and Methods

Peptide synthesis

Peptides used in this study were synthesized using an automated synthesizer (AAPPTEC Focus-XC-6V instrument running AAPPTEC software version of 3.02.03). The sequences were assembled starting from preloaded amino acid on 2-chlorotrityl resins. Standard fluorenylmethoxycarbonyl (Fmoc) chemistry was used to construct the required sequences. For coupling, 8.0 eq. of suitably protected amino acids (0.4 M in dimethylformamide [DMF]) was activated with *N,N,N′,N′*-tetramethyl-*O*-(1*H*-benzotriazol-1-yl)uronium hexafluorophosphate (0.4 M in DMF, 8.0 eq.) and *N*-methylmorpholine (NMM, 0.8 M, 16.0 eq.) and transferred to the peptide vessels under positive pressure of nitrogen. After a coupling time of 60 min, the reagents were removed and resins were washed with DMF. Fmoc-protecting groups were removed with 20% piperidine in DMF, washed with DMF, and taken to next coupling with the respective amino acid.

Peptide cleavage

Procedure A (protected sequences, except the N- and C-terminus). After the final amino acid was added to the resins, Fmoc-protecting groups of *N*-termini were removed by 20% piperidine in DMF (2 × mL, 15.0 min each), washed with DMF, and transferred to another manual synthesis peptide vessel. The resins were washed with dichloromethane (DCM) (3 × mL) and drained under positive pressure of nitrogen. A cleavage cocktail consisting of 6:2:2 DCM:acetic acid (HOAc):2,2,2-trifluoroethanol was added to the resins (15.0 mL/g of the resin) and the vessels were shaken for 60 min and drained under positive pressure of nitrogen. This procedure was repeated three more times and the combined filtrates were concentrated under reduced pressure. Any remaining HOAc was removed azeotropically with heptane (3 × 50 mL). The resulting paste was diluted with water to precipitate the protected peptides, washed with water, and dried under vacuum before being taken to conjugation with dyes.

Procedure B (native sequences). A cleavage cocktail of 95:2.5:2.5 trifluoroacetic acid:water:triisopropylsilane (15.0 mL/g) was added to the resins and the vessels were shaken for 30 min. The solution was filtered, and the resins were washed two more times with the cleavage solution. The combined filtrates were concentrated under reduced pressure and purified by preparative high-performance liquid chromatography (HPLC).

Procedure C. The crude peptide was dissolved in 98:2:1 TAA/water/phenol (v/v/v; 5.0 mL) and stirred for 60 min at room temperature. All the volatiles were evaporated under reduced pressure and the residue was purified by preparative HPLC.

Conjugation of sulfo-cyanine 5 *N*-hydroxysuccinimidyl ester to the peptides. Fully protected peptide amines cleaved from the resin (Procedure A, 0.01 mmol) were dissolved in DMF (1.0 mL) and the pH was adjusted to 8.0–9.0 with NMM. Sulfo-Cyanine 5 *N*-hydroxysuccinimidyl ester (Lumiprobe, Hunt Valley, MD; 8.0 mg, 0.0102 mmol) was added as solid and stirred at 45°C for 12 h under argon protected from light. DMF was removed under reduced pressure; the residues were deprotected (procedure C) and purified by HPLC.

All the sequences used in this study were purified and analyzed by the following conditions:

Preparative HPLC. Column—30.0 × 150 mm Waters XBridge® C18; Particle size 5.0 μm; Solvent A: water with 0.1% TFA; Solvent B: acetonitrile with 0.1% TFA; Elution rate: 30.0 mL/min; Detection @ 220 nm; and Gradient: 10% B to 80% B over 60 min. Major peaks were collected and analyzed by liquid chromatography/mass spectrometry. Fractions with the required mass and purity of ≥95% were pooled and freeze dried to yield the final peptides as fluffy solids.

Analytical HPLC conditions. Column—Agilent Zobrax C18; 4.6 mm × 50 mm; 3.5 μm; Solvent A: water with 0.05% TFA and Solvent B: acetonitrile with 0.05% TFA; Elution rate: 1.0 mL/min; Detection @ 220 nm; and Gradient: 5% B to 95% B over 7 min.
